# Altered Cardiac Electrophysiology and SUDEP in a Model of Dravet Syndrome

**DOI:** 10.1371/journal.pone.0077843

**Published:** 2013-10-14

**Authors:** David S. Auerbach, Julie Jones, Brittany C. Clawson, James Offord, Guy M. Lenk, Ikuo Ogiwara, Kazuhiro Yamakawa, Miriam H. Meisler, Jack M. Parent, Lori L. Isom

**Affiliations:** 1 Department of Pharmacology, University of Michigan, Ann Arbor, Michigan, United States of America; 2 Department of Human Genetics, University of Michigan, Ann Arbor, Michigan, United States of America; 3 Department of Neurology, University of Michigan, Ann Arbor, Michigan, United States of America; 4 Molecular and Integrative Physiology, University of Michigan, Ann Arbor, Michigan, United States of America; 5 Veterans Affairs Ann Arbor Healthcare System, Ann Arbor, Michigan, United States of America; 6 Laboratory for Neurogenetics, Riken Brain Science Institute, Wako, Japan; University of Milan, Italy

## Abstract

**Objective:**

Dravet syndrome is a severe form of intractable pediatric epilepsy with a high incidence of SUDEP: Sudden Unexpected Death in epilepsy. Cardiac arrhythmias are a proposed cause for some cases of SUDEP, yet the susceptibility and potential mechanism of arrhythmogenesis in Dravet syndrome remain unknown. The majority of Dravet syndrome patients have *de* *novo* mutations in *SCN1A*, resulting in haploinsufficiency. We propose that, in addition to neuronal hyperexcitability, *SCN1A* haploinsufficiency alters cardiac electrical function and produces arrhythmias, providing a potential mechanism for SUDEP.

**Methods:**

Postnatal day 15-21 heterozygous *SCN1A-R1407X* knock-in mice, expressing a human Dravet syndrome mutation, were used to investigate a possible cardiac phenotype. A combination of single cell electrophysiology and *in* *vivo* electrocardiogram (ECG) recordings were performed.

**Results:**

We observed a 2-fold increase in both transient and persistent Na^+^ current density in isolated Dravet syndrome ventricular myocytes that resulted from increased activity of a tetrodotoxin-resistant Na^+^ current, likely Na_v_1.5. Dravet syndrome myocytes exhibited increased excitability, action potential duration prolongation, and triggered activity. Continuous radiotelemetric ECG recordings showed QT prolongation, ventricular ectopic foci, idioventricular rhythms, beat-to-beat variability, ventricular fibrillation, and focal bradycardia. Spontaneous deaths were recorded in 2 DS mice, and a third became moribund and required euthanasia.

**Interpretation:**

These data from single cell and whole animal experiments suggest that altered cardiac electrical function in Dravet syndrome may contribute to the susceptibility for arrhythmogenesis and SUDEP. These mechanistic insights may lead to critical risk assessment and intervention in human patients.

## Introduction

Dravet Syndrome (DS, previously known as Severe Myoclonic Epilepsy of Infancy) is a devastating, intractable pediatric epileptic encephalopathy [1]. Patients exhibit developmental regression during early childhood and frequent pharmacoresistant seizures [1-3]. Up to 15% of DS subjects die during early childhood or adolescence, and most of these cases are thought to represent SUDEP [2], defined as Sudden, Unexpected, witnessed or unwitnessed, nontraumatic and nondrowning Death in patients with EPilepsy [4], excluding cases of documented status epilepticus. In the most widely used definition, death may occur with or without evidence of a seizure, and postmortem examination does not reveal a toxicological or anatomical cause of death. SUDEP accounts for 7.5-17% of all deaths in epilepsy [5,6]. Indirect evidence has linked SUDEP to seizure-induced apnea, pulmonary edema, dysregulation of cerebral circulation, and cardiac arrhythmias [5-7]. Arrhythmias may also occur secondary to hormonal or metabolic changes or autonomic discharges [6-8]. 

More than 80% of DS patients carry *de novo* mutations in *SCN1A* [9,10] that result in haploinsufficiency. *SCN1A* encodes the voltage-gated Na^+^ channel (VGSC) α subunit Na_v_1.1. The tetrodotoxin–resistant (TTX-R) Na_v_1.5 channel, encoded by *SCN5A*, is the predominant VGSC in the mammalian heart [11]. TTX-sensitive (TTX-S) VGSCs, including Na_v_1.1, Na_v_1.3, and Na_v_1.6, are also expressed in areas of the heart that include the ventricles and sino-atrial node, although their function is not well understood [11-16]. More recently, Kaufmann and colleagues [17] showed that, in addition to Na_v_1.5, human atrial myocytes express TTX-S VGSCs Na_v_1.1, Na_v_1.2, Na_v_1.4, and Na_v_1.6. VGSCs provide a pore for the movement of Na^+^ into the cell, resulting in a rapidly activating and inactivating transmembrane current (I_Na_) responsible for the action potential (AP) upstroke and impulse propagation. The level of functional expression and biophysical properties of ion channels give the cardiac AP its characteristic shape. The balance between depolarizing currents (e.g., I_Na_ and I_CaL_) and repolarizing currents (e.g., I_to_, I_Kr_, I_Ks_, and I_K1_) determines the level of excitability, AP morphology, AP duration (APD), and dynamics of impulse propagation [18]. Channelopathies disrupt this balance, leading to AP changes and increased susceptibility to arrhythmias and sudden death [10,19]. Cardiac arrhythmogenic diseases can result from gain-of-function (e.g., Long QT Syndrome-3, LQTS-3) or loss-of-function (e.g., Brugada Syndrome) mutations in *SCN5A* [20]. LQTS-3 mutations result in increased persistent I_Na_ during the AP plateau, leading to triggered activity in the form of early after-depolarizations (EADs), and providing a substrate for arrhythmogenesis [19]. Homozygous deletion of *Scn1a*, *Scn2a*, *Scn5a*, or *Scn8a* in mice is lethal, revealing their non-redundant functions [10,21-23]. Blockade of TTX-S VGSCs in the heart results in altered heart rate (HR) and cardiac contractility [11,12,14,17,24]. Despite many studies examining the effects of *SCN1A* mutations in the nervous system [3], the influence of *SCN1A* abnormalities on cardiac function remains unknown. 

We propose that the strong association between epilepsy and SUDEP in DS subjects is a consequence of expression of mutant *SCN1A* in both brain and heart. Recent work, using a *Scn1a* ^*+/-*^ DS mouse model as well as an inhibitory neuron-specific *Scn1a* ^*+/-*^ line, suggested that SUDEP may be caused by parasympathetic hyperactivity immediately following seizures, leading to atrioventricular nodal block and lethal bradycardia [25]. While this study implicated cardiac dysfunction in DS-linked SUDEP, the excitability of individual cardiac myoyctes was not investigated. Further, instead of studying the effects of spontaneous seizures, as proposed to occur in SUDEP patients, this study utilized acute hyperthermia-induced seizures. Our objective here was to fill a critical gap in the literature by determining whether cardiac myocytes isolated from mice expressing a human *SCN1A* DS mutation [26] have altered excitability and whether DS mice exhibit cardiac dysfunction following spontaneous seizures. We propose that, in addition to neuronal dysfunction, *Scn1a* haploinsufficiency produces altered cardiac electrical function and arrhythmias, providing a cardiac contribution to the mechanism of SUDEP. We report that *Scn1a-R1407X* heterozygous mice have increased TTX-R, but not TTX-S, cardiac I_Na_, as well as altered AP and ECG properties, EADs, and arrhythmias that produce SUDEP-like events. Our results provide novel insights into an ion channelopathy that provides critical conditions for arrhythmogenesis, and suggest a mechanism for SUDEP that includes changes in cardiac I_Na_. 

## Materials and Methods

### Animals

*SCN1A* ^*R1407X/+*^ mice, previously maintained on the C57BL/6J background [26], were backcrossed to C3HFeB/HeJ (Jackson Laboratory, Bar Harbor, ME) to increase litter size. Heterozygous mutant mice of both genders from the N3 and N4 generations were studied at postnatal day (P)15-21. Whenever possible, all data analysis was conducted blinded to genotype. Heterozygous *Scn1a* ^*R1407X/+*^ mice are designated DS throughout the manuscript.

### Ethics Statement

This study was carried out in strict accordance with the recommendations in the Guide for the Care and Use of Laboratory Animals of the National Institutes of Health. The protocol was approved by the University Committee on the Use and Care of Animals at the University of Michigan (Approval Numbers: 04695 and 09790). All efforts were made to minimize suffering. 

### Genotyping

The R1407X mutation abolishes a *Hpa*II restriction site in the wildtype *Scn1a* sequence. DS mice were genotyped by PCR amplification of a 518 bp genomic fragment with the primers DS-F (5’ CAATGATTCCTAGGGGGATGTC 3’) and DS-R (5’ GTTCTGTGCACTTATCTGGATTCAC 3’). Digestion of the PCR product with HpaII generated 2 fragments, 295 and 223 bp, from the wildtype allele and an uncut 518 bp fragment from the mutant allele. Genomic DNA was amplified in a 25 µl reaction containing 1X GoTaq Buffer, 0.2 mM dNTPs, 0.5 µM each primer, 1 unit GoTaq DNA Polymerase (Promega). Incubation at 94°C for 3 min was followed by 31 cycles of 94°C for 30 sec, 62°C for 1 min, 72°C for 1 min followed by incubation at 72°C for 6 min. After digestion with *Hpa*II, PCR products were separated on 2% agarose gels containing 0.15 µg/ml ethidium bromide. 

### Acutely Isolated Adult Mouse Ventricular Myocytes

WT and DS cardiac myocytes were acutely isolated from P15–21 mice using a protocol modified from Cerrone et al. [27]. In brief, hearts were isolated from WT and DS mice, and placed in ice-cold perfusion buffer. The Ca^2+^ free perfusion buffer consisted of (mM): 10 HEPES, 0.6 Na_2_HPO_4_, 113 NaCl, 4.7 KCl, 12 NaHCO_3_, 0.6 KH_2_PO_4_, 1.2 MgSO_4_, 10 KHCO_3_, 30 Taurine, 5.5 glucose, and 10 butaneodione monoxime. The hearts were cannulated and cleared with perfusion buffer (37°C, 3 ml/min). Next, type-II collagenase (0.87 mg/ml, Worthington Biochemical), trypsin (0.14 mg/ml), and 12 µM CaCl_2_ were added to the perfusion buffer for the enzymatic digestion. The lower two-thirds of the hearts were isolated and minced into small pieces in digestion buffer. The digestion reaction was stopped by resuspension in stopping buffer, which included perfusion buffer plus 10% fetal bovine serum and 12.5 µM CaCl_2_. The solution was then incrementally brought up to 1 mM CaCl_2_. Healthy ventricular cardiac myocytes were defined as those that were Ca^2+^ tolerant, rod shaped, striated, and quiescent, with a resting membrane potential less than or equal to -65 mV. All myocyte recordings were acquired within 8 h of the cell isolation.

### Single Cell Electrophysiology

Standard voltage and current clamp techniques were used to assess the effects of DS mutatons on cardiac I_Na_ and AP properties, respectively [28,29]. Single cell cardiac electrophysiological properties were acquired from healthy cardiac myocytes. Experiments were performed using borosilicate glass pipettes with resistance of <3MΩ for I_Na_ and 4-5 MΩ for AP recordings. Data were acquired using an Axopatch 200B amplifier (Molecular Devices, USA). The data were acquired and analyzed using pCLAMP9-10 (Molecular Devices, USA) and custom AP analysis software (National Instruments LabView, USA)

### Voltage Clamp Recordings

Voltage clamp I_Na_ recordings were performed at room temperature (21–22°C) with 5 mM [Na^+^]_o_. The extracellular solution contained (in mM): 5 NaCl, 1 MgCl_2_, 1 CaCl_2_, 0.1 CdCl_2_, 11 Glucose, 132.5 CsCl, and 20 HEPES. The filling solution contained (in mM): 5 NaCl, 135 CsF, 10 EGTA, 5 MgATP, and 5 HEPES. Upon gaining access to the cell, appropriate whole cell and series resistance compensation (<70%) and leak subtraction were applied. Whole cell I_Na_, TTX-R I_Na_, TTX-S I_Na_ density and biophysical properties were assessed. Assessment of transient and persistent I_Na_ density, I_Na_ inactivation time, and the voltage dependence of I_Na_ conductance were obtained by holding the cell at -120 mV, followed by stepping to voltages between -100 and +30 mV, in 5 mV steps, for 200 ms, with 2800 ms interpulse intervals. The voltage dependence of I_Na_ availability was determined by holding at various voltages (-160 mV to 0 mV, 5 mV increments, 200 ms duration) and stepping to -40 mV (30 ms), with 2770 ms interpulse intervals at -120 mV. The normalized voltage dependence of I_Na_ availability and conductance (based upon each cells’ reversal potential) were fit to a Boltzmann function, and differences in the V_½_ and slope factor were compared between groups. The time dependence of I_Na_ recovery was assessed by holding at -120 mV and stepping to -30 mV for 20 ms (P1), followed by a 1–40 ms (1 ms increments) interpulse interval at -120 mV, and a second step to -30 mV for 20 ms (P2). The time dependence of I_Na_ recovery was calculated by P2/P1 at each timepoint, and these results were fit to a single exponential function. The rate of I_Na_ inactivation was fit to a double exponential function. 100 nM TTX was added to block only the TTX-S I_Na_, and therefore pharmacologically separate the TTX-R and TTX-S I_Na_. TTX (30 µM) was then given to block all I_Na_, and used for measuring the persistent I_Na_ (pre- minus post-30 µM TTX). The persistent current was measured 30-35 ms after the voltage step, which was a time when the current amplitude was stable. These persistent I_Na_ results were also confirmed using the P/4 method, yielding similar results. 

### Current Clamp Recordings

Current clamp AP recordings were acquired at 37°C in standard Tyrodes solution (in mM): 148 NaCl, 0.4 NaH_2_PO_4_, 1 MgCl_2_, 5.4 KCl, 1 CaCl_2_, 5.5 glucose, 15 HEPES. The internal solution included (in mM): 148 KCl, 1 MgCl_2_, 5 EGTA, 5 HEPES, 2 creatine, 5 K_2_-ATP, 5 phosphocreatine. Incremental amounts of current (0.1 nA steps, 0.3 ms) and pacing cycle lengths (2000 ms, 1000 ms, and up to the fastest pacing cycle length indicated, in 1 hertz (Hz) increments) were used to assess changes in excitability, AP morphology, and susceptibility to triggered activity (i.e. EADs). Only cells with a diastolic membrane potential more negative than -65 mV were used for analysis.

### In Vivo ECG Recordings

P15-17 WT (N = 8) and DS (N = 13) mice were implanted with radiotelemetry ECG devices (DSI ETA-F10) at the University of Michigan Phenotyping Core. Animals were anesthetized (isoflurane) and the unit was implanted on the dorsal surface via a small 1 cm incision. Next, a small midline incision was made from the xiphoid process to the manubrium. The leads were passed over the shoulder subcutaneously and sutured onto the intercostal muscles of the rib cage, for ECG lead configuration II. The incisions were sutured closed, the animals were treated with prophylactic antibiotics, and all mice successfully recovered from the procedure without any signs of complications. The first WT mouse with a non-working test unit remained viable without any pathologies (>180 days). ECG (1 KHz sampling), temperature, activity, and running wheel activity were acquired continuously until P70 to provide mechanistic insights into the DS *in vivo* cardiac phenotype and the events precipitating SUDEP. Mice were housed in a temperature controlled room (21°C) in separate cages on a 12 h light-dark cycle (6 AM - 6 PM). Recordings were monitored and analyzed remotely. The alterations in the ECG waveform were documented during pentylenetetrazole-induced convulsive seizures (PTZ, 40 mg/kg loading and then 20 mg/kg repeated every 20 min intraperitoneally) and the period leading up to spontaneous death in non-PTZ treated mice. All surviving animals were euthanized at the end of the study.

### Quantitative Reverse Transcriptase-Polymerase Chain Reactions (qRT-PCR)

Total RNA was isolated from individual hearts using Trizol Reagent (Abion/RNA). Aliquots (1.5 ug) of total RNA were treated with DNAse I (Invitrogen) and cDNA was prepared using the SuperScript II First Strand Synthesis System for RT-PCR (Invitrogen). The *Scn5a* transcript was quantified using TaqMan gene expression assays Mm01342518_m1 and Mm00451971_m1 (ABI) which span introns 7 and 12, respectively. As an internal control, the 18S transcript was quantified using TaqMan assay Mm03928990_g1. Fluorescence was measured on a Step 1 Real Time PCR System (ABI) at the Microarray Core at the University of Michigan. The mean C_T_ value was determined from quadruplicate assays of each sample. The value of ΔC_T_ was calculated by subtracting the C_T_ for 18S from the C_T_ for Scn5a. 

### Western Blot Analysis

Western blot analysis was performed as previously described [30]. Anti-Na_v_1.5 antibody was provided by Dr. Peter Mohler [31] and used at a dilution of 1:1000. Secondary antibody was goat anti-rabbit antibody conjugated to horseradish peroxidase used at a dilution of 1:800 (Thermo Scientific). Signals were visualized using a chemiluminescence system (Supersignal West Femto Maximum Sensitivity Substrate, Thermo Scientific), detected on a Li-Cor Odyssey using ImageStudio software.(Li-Cor).

### Statistical Analyses

Results are expressed as mean ± standard error of the mean. Unpaired t test with Welch’s correction, χ^2^ Test and Log-rank, Mantel-Cox, Survival Test were used as appropriate to test for significance between genotypes, and significance was considered as *p* ≤ 0.05.

## Results

### DS mice have increased TTX-R transient and persistent I_Na_ density

**Figure 1:**
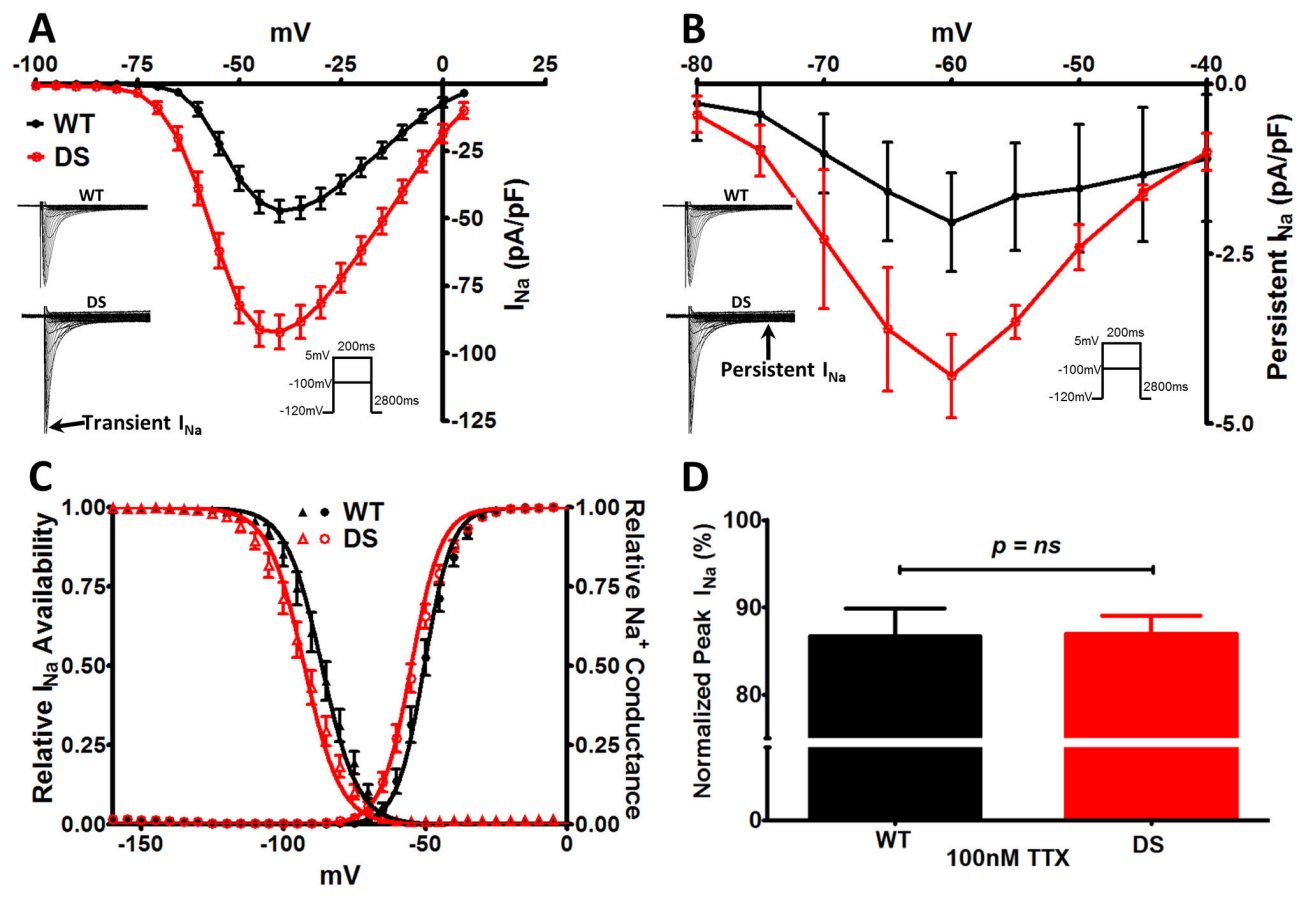
DS Mice Have Altered Cardiac I_Na_ Properties. A. Current-voltage (I-V) relationship of transient I_Na_. Peak transient I_Na_ density is increased 2-fold in the DS (N = 6, n = 14) vs WT cardiac myocytes (N = 8, n = 20, *p* < 0.0001). *Inset*: Representative traces from each group. B. I-V relationship for persistent I_Na_ (pre- minus post-30 µM TTX) also shows a 2-fold increase in peak persistent I_Na_ in the DS vs. WT groups. To further confirm these results we employed the P/4 method to measure the persistent I_Na_, yielding similar results (-60 mV, WT, -1.72 ± 0.50; DS, -3.88 ± 0.72, N = 2, n = 5-9, *p* = 0.02). C. Leftward shift (V_½_ of Boltzman fit, *p* = 0.04) in the voltage dependence of I_Na_ availability and conductance in the DS group. D. Similar percent change in peak transient I_Na_ density upon administration of 100 nM TTX in the WT and DS groups. Unpaired t-test with Welch’s correction.

To determine whether DS mice have altered cardiac excitability, we examined the I_Na_ properties of acutely isolated ventricular myocytes. We reported previously that *Scn1a* haploinsufficiency results in *increased*, rather than decreased, I_Na_ density and hyperexcitability in DS patient-specific induced pluripotent stem cell (iPSC)-derived neurons [32]. Consistent with this, we observed a 2-fold increase in the peak transient (p < 0.0001) and persistent (p ≤ 0.05) I_Na_ density in cardiac myocytes from DS vs. WT littermates (Figure **1** **, A and B**), with a hyperpolarizing shift in the voltage dependence of I_Na_ availability and conductance (Figure **1 C**, p ≤ 0.05). Since the voltage dependence for pure or predominantly TTX-R Na_v_1.5 expressing cells is more negative compared to TTX-S (e.g. Na_v_1.1, Na_v_1.3, or Na_v_1.6) VGSC expressing cells, these results suggested a change in the proportion of total cellular I_Na_ carried by Na_v_1.5 [28,32-41]. We administered 100 nM TTX to test for potential changes in functional TTX-S I_Na_ density in the DS cardiac myocytes. We observed a similar reduction in DS and WT cells (Figure **1 D**), consistent with previous reports [42,43]. These data suggest that the observed increase in I_Na_ in DS cardiac myocytes was not due to increased functional TTX-S VGSC expression and was instead due to an increase in activity of the predominant cardiac TTX-R VGSC, Na_v_1.5. 

**Figure 2:**
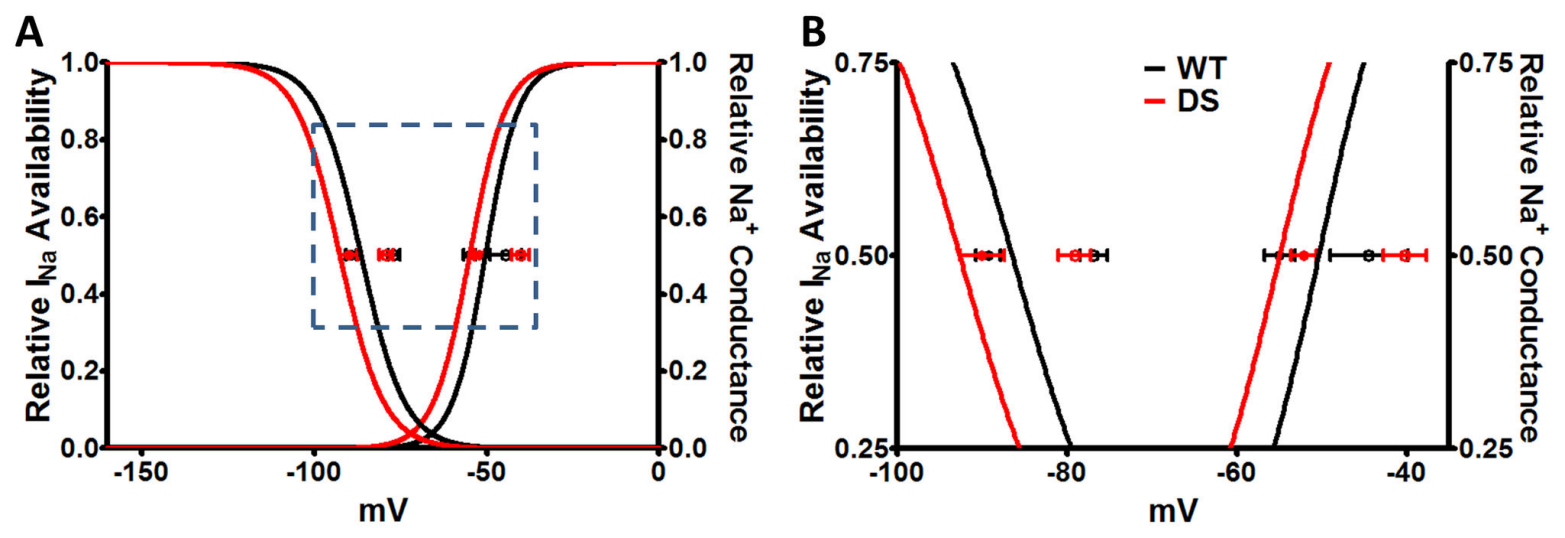
Isolation of TTX-R and TTX-S I_Na_ Biophysical Properties. A. Boltzman curves for the voltage dependence of I_Na_ availability and conductance for the total cardiac I_Na_ (TTX-S + TTX-R I_Na_; reproduction of the curve-fits from Figure 1C). In both WT and DS myocytes the V_½_ values of TTX-R I_Na_ (closed circles, following blockade of TTX-S I_Na_ with 100 nM TTX) and TTX-S I_Na_ (open circles, defined as total I_Na_ minus TTX-R I_Na_) are plotted. Pharmacological separation of TTX-S and TTX-R I_Na_ was confirmed by the loss of difference in the V_½_ values between WT vs DS, and the development of a significant difference between the TTX-S vs. TTX-R V ½ values for I_Na_ availability and conductance. B. Zoom-in of the boxed region in A.

We used 100 nM TTX to pharmacologically isolate the TTX-R and TTX-S I_Na_. Regardless of genotype, and as expected, the V_½_ values for TTX-R I_Na_ (following blockade of TTX-S I_Na_ with 100 nM TTX) vs. TTX-S I_Na_ (defined as total I_Na_ minus TTX-R I_Na_) were significantly different. Figure **2** illustrates that pharmacological isolation of each current led to the expected shifts between TTX-R vs. TTX-S I_Na_ voltage dependent properties (p ≤ 0.05), with V_½_ values for TTX-R I_Na_ being more negative than TTX-S I_Na_ availability and conductance. However, a comparison of V_½_ values for the pharmacologically separated currents between genotypes showed no differences, suggesting that the observed increases in total I_Na_ were due to increases in the level of functional channel expression rather than changes in voltage-dependence. 

Table **1** shows a detailed biophysical characterization of I_Na_ properties. No changes were observed in the voltage of peak I_Na_, I_Na_ reversal potential, or normalized I_Na_-voltage relationships between genotypes. While the slope factors for the voltage dependence of the total I_Na_ availability and conductance did not differ between genotypes (Figure **1C**, Table **1**), when we pharmacologically separated the TTX-S and TTX-R I_Na_ (Figure **2**, Table **1**), we observed slope factor differences. The slope factors for TTX-R Na^+^ conductance and TTX-S I_Na_ availability were significantly increased in DS, suggesting changes in the sensitivity of these channels to changes in voltage.

**Table 1:** I_Na_ biophysical Properties in Each Group.

	***Mouse***		
**Parameter**	**WT**	**DS**	
**N=mice, n=cells**	N=5-6, n=10-14	N=6-8, n=14-20	
**Capacitance (pF)**	84.0 ± 6.4	61.0 ± 3.5	*p*=0.005
**Peak I_Na_ (pA/pF)**	-48.6 ± 4.0	-94.8 ± 6.3	*p*<0.0001
**V of Peak I_Na_ (mV)**	-40.4 ± 1.0	-42.5 ± 1.2	*p*=0.17
**I_Na_ Rev. Pot. (mV)**	5.1 ± 1.9	9.2 ± 1.5	*p*=0.09
**I_Na_ Availability**			
V ½	-86.5 ± 1.8	-92.6 ± 1.8	*p*=0.02
Slope Factor	6.3 ± 0.2	6.4 ± 0.1	*p*=0.81
**I_Na_ Conductance**			
V ½	-50.4 ± 1.3	-55.0 ± 1.4	*p*=0.02
Slope Factor	4.9 ± 0.2	5.3 ± 0.3	*p*=0.19
**I_Na_ Decay (τ_fast_)**			
-50	2.4 ± 0.3	1.7 ± 0.1	*p*=0.02
-45	1.8 ± 0.1	1.4 ± 0.1	*p*=0.04
-40	1.3 ± 0.1	1.2 ± 0.1	*p*=0.31
-35	1.2 ± 0.1	1.0 ± 0.1	*p*=0.03
-30	1.1 ± 0.1	0.8 ± 0.1	*p*=0.01
**I_Na_ Decay (τ_slow_)**			
-50	5.2 ± 0.8	5.9 ± 0.7	*p*=0.50
-45	4.1 ± 0.5	4.5 ± 0.4	*p*=0.59
-40	4.1 ± 0.5	3.8 ± 0.4	*p*=0.59
-35	2.5 ± 0.3	3.0 ± 0.3	*p*=0.32
-30	2.1 ± 0.3	2.6 ± 0.3	*p*=0.28
**I_Na_ Recovery (tau)**	4.0 ± 0.4	4.8 ± 0.4	*p*=0.19
**Persistent I_Na_ @** ………. **-60 millivolts (pA/pF**)	-2.0 ± 0.7	-4.3 ± 0.6	*p*=0.04
	(N=2; n=7)	(N=1; n=5)	
**TTX Resistant I_Na_ (%)**	86.7 ± 3.1	87.0 ± 2.1	*p*=0.94
	(N=2; n=7)	(N=1; n=5)	
**TTX-Resistant I_Na_ Availability**			
V ½	-89.3 ± 1.4	-90.0 ± 2.6	*p*=0.81
Slope Factor	6.2 ± 0.3	7.2 ± 0.5	*p*=0.11
**TTX Resistant I_Na_ Conductance**			
V ½	-55.0 ± 1.7	-52.1 ± 1.5	*p*=0.24
Slope Factor	4.7 ± 0.4	6.1 ± 0.4	*p*=0.04
**TTX-Sensitive I_Na_ Availability**			
V ½	-76.9 ± 1.6	-79.1 ± 1.9	*p*=0.40
Slope Factor	4.1 ± 0.3	5.2 ± 0.2	*p*=0.01
**TTX Sensitive I_Na_ Conductance**			
V ½	-44.5 ± 4.6	-40.2 ± 2.5	*p*=0.43
Slope Factor	4.7 ± 1.0	6.5 ± 0.5	*p*=0.15

Significant differences between the WT vs. DS myocytes are defined as *p* ≤ 0.05.

### Differences in Scn5a transcription and translation are undetectable

**Figure 3:**
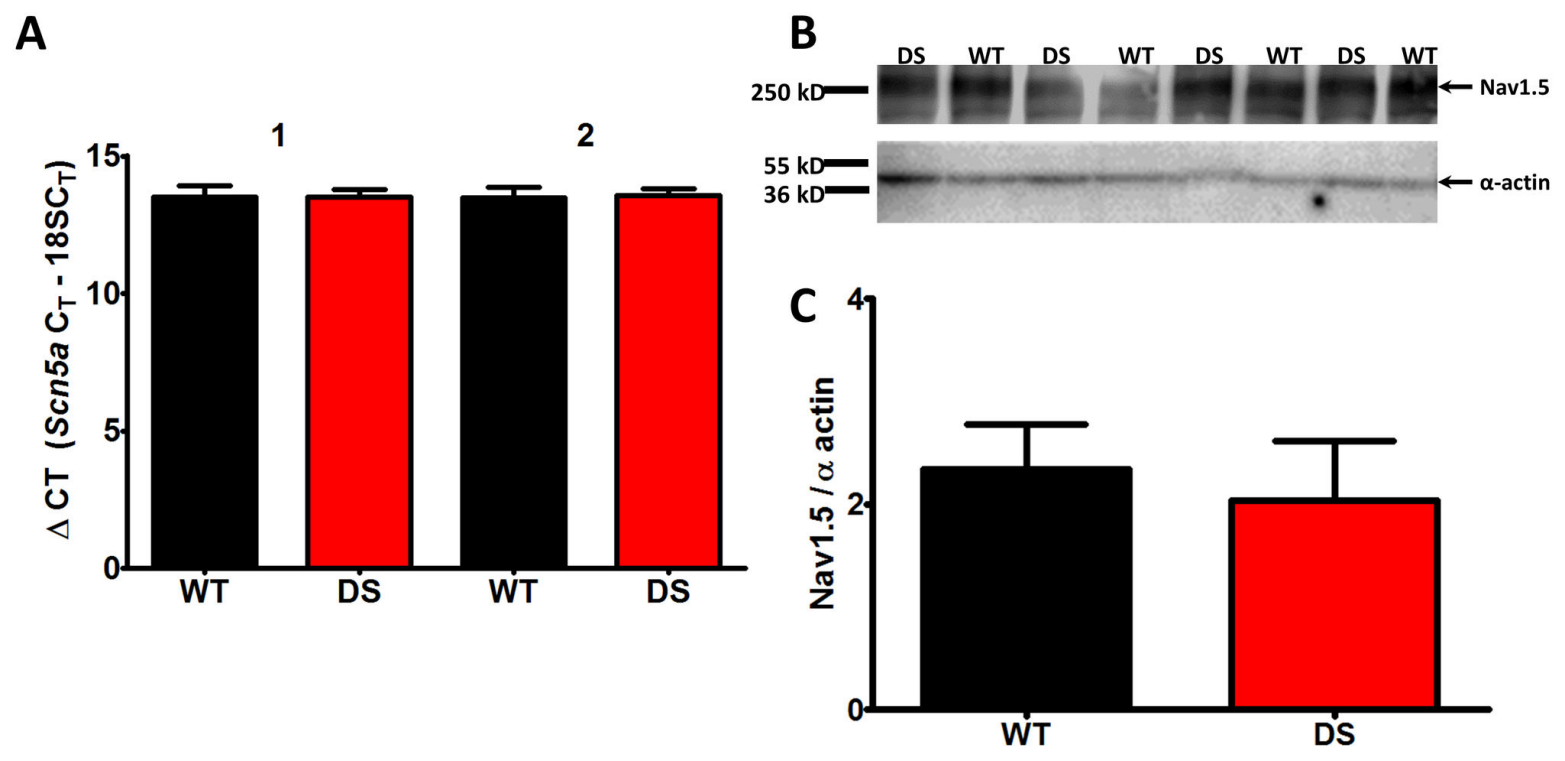
mScn5a and Nav1.5 levels are unchanged in DS mutant hearts. A. Heart RNA from biological replicates (DS mice, n = 4; WT mice, n = 5) were used to generate two independent cDNAs per animal. The cDNAs were assayed using qPCR in quadruplicate with two independent *Scn5a* TaqMan primer sets and normalized to 18s RNA. B. Western blots of membrane proteins isolated from DS and WT ventricular CMs. 50 µg of protein was loaded in each lane, and probed with anti-Na_v_1.5 (Mohler 1:1000), and anti-α-actin (Sigma 1:500), which served as the loading control. C. Quantification of Na_v_1.5 expression normalized to α-actin expression.

DS cardiac myocytes exhibited a 2-fold increase in TTX-R transient and persistent I_Na_ density compared to WT myocytes. In an effort to understand the molecular mechanism for this difference, we performed quantitative RT-PCR (qRT-PCR) to ascertain whether we could detect differences in the level of *Scn5a* transcripts between genotypes. Using two different *Scn5a* primer pairs and two independent cDNAs per animal (n=4-5), we observed no change in the amount of *Scn5a* transcript (Figure **3A**). To assess differences in channel protein expression, we quantified Na_v_1.5 polypeptide levels in membrane enriched ventricular myocyte fractions from each genotype. As Figure **3B-C** indicate, we found no measurable changes in Na_v_1.5 expression, suggesting a post-translational mechanism, e.g. altered channel trafficking, phosphorylation, or association with cytoskeletal proteins in specific subcellular domains.

### DS cardiac myocytes are hyperexcitable

**Figure 4:**
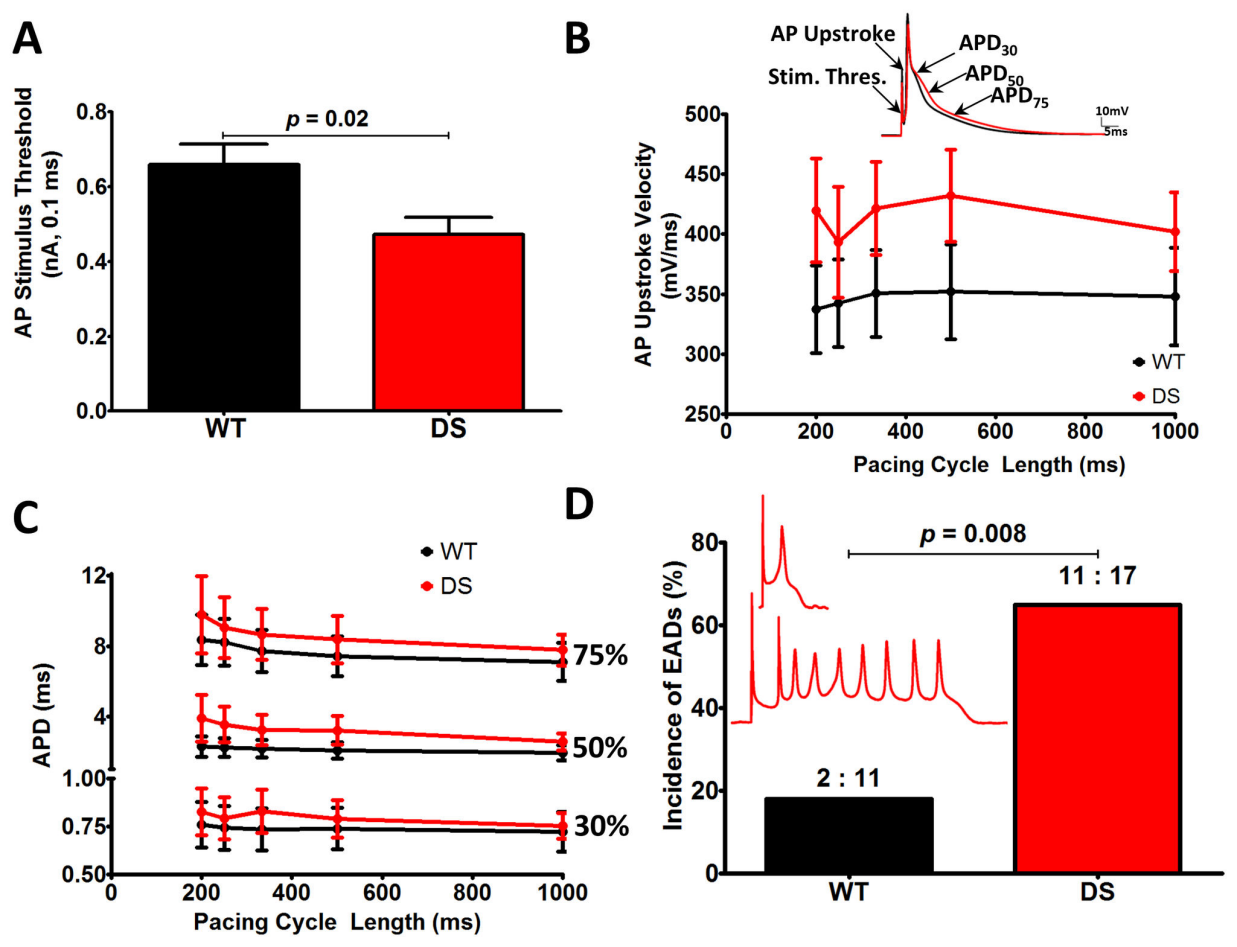
DS myocytes exhibit increased excitability and incidence of early after depolarizations (EADs). A. DS myocytes require significantly less injected current to fire APs. B. DS myocyte AP upstroke velocity is faster at all pacing cycle lengths (*p* = ns). C. Slight prolongation of the AP duration at 30%, 50%, and 75% repolarization at many pacing cycle lengths (*p* = ns). D. DS myocytes are significantly more susceptible to EADs, a substrate for arrhythmogenesis. *Inset*: Representative EADs from DS myocytes (red.) Panels A-C, unpaired t-test with Welch’s correction. Panel D χ^2^ Test (WT, N = 9, n = 11, DS, N = 8, n = 17).

To determine whether the observed changes in I_Na_ resulted in altered cardiac myocyte excitability, we recorded APs from isolated WT and DS ventricular cardiac myocytes. DS cardiac myocytes were hyperexcitable compared to WT. DS cells required significantly less current to initate AP firing (*p* ≤ 0.05, Figure **4A**). At all pacing cycle lengths, WT and DS mice had similar diastolic membrane potentials (ranging between -75 mV to -72 mV). At each pacing cycle length, we observed non-significant trends for increased AP upstroke velocity and APD in DS vs. WT cardiac myocytes (Figure **4B and C**). Despite these values not reaching statistical significance, we observed a significant increase in the incidence of EADs in DS (67%) vs. WT cardiac myocytes (18%, *p* ≤ 0.05, Figure **4D**), which provides a substrate for the initiation of cardiac arrhythmias [19]. 

### DS leads to changes in cardiac excitability and sudden death

**Figure 5:**
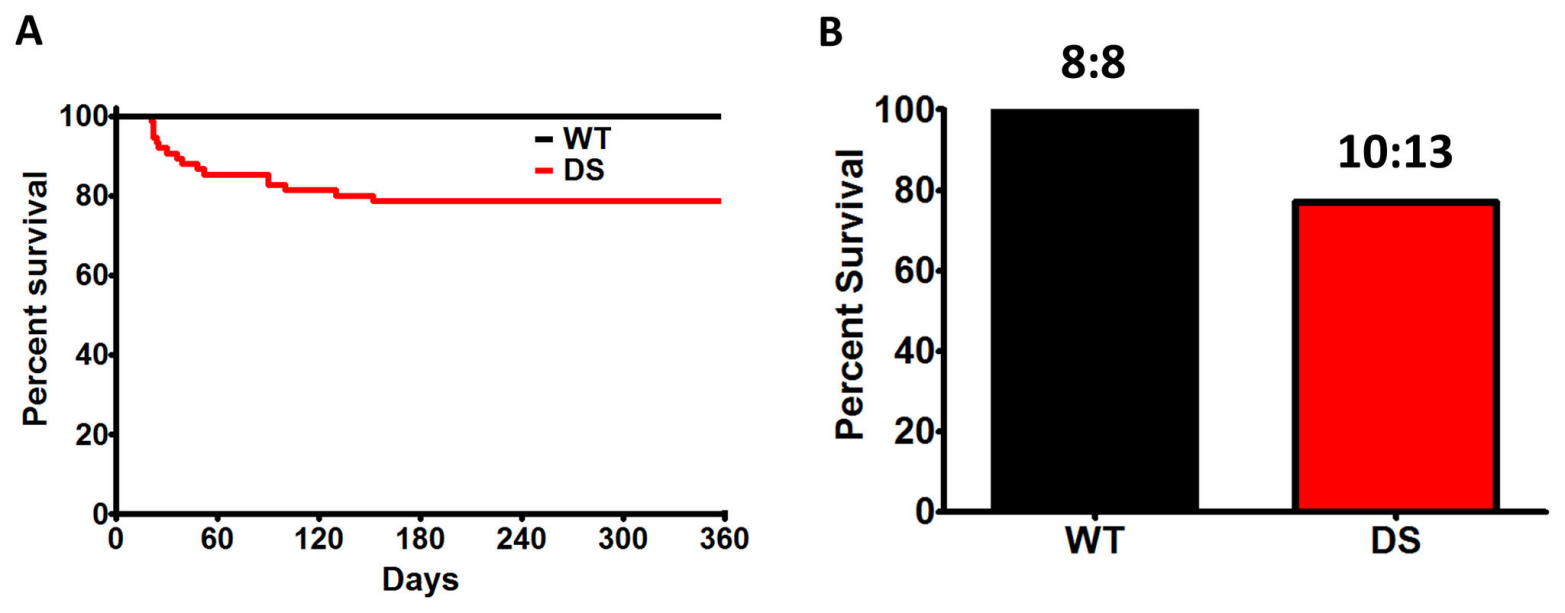
DS Mice Undergo SUDEP. A. Kaplan-Meier survival curves for WT and DS mice (N = 75 for each group, p < 0.0001, Log-rank, Mantel-Cox, Survival Test). B. Percent survival in WT (N = 8) and DS (N = 13) mice implanted with radiotelemetry units. SUDEP or near-SUDEP in 3 DS mice (at P41, P45, and P51, respectively).

**Figure 6:**
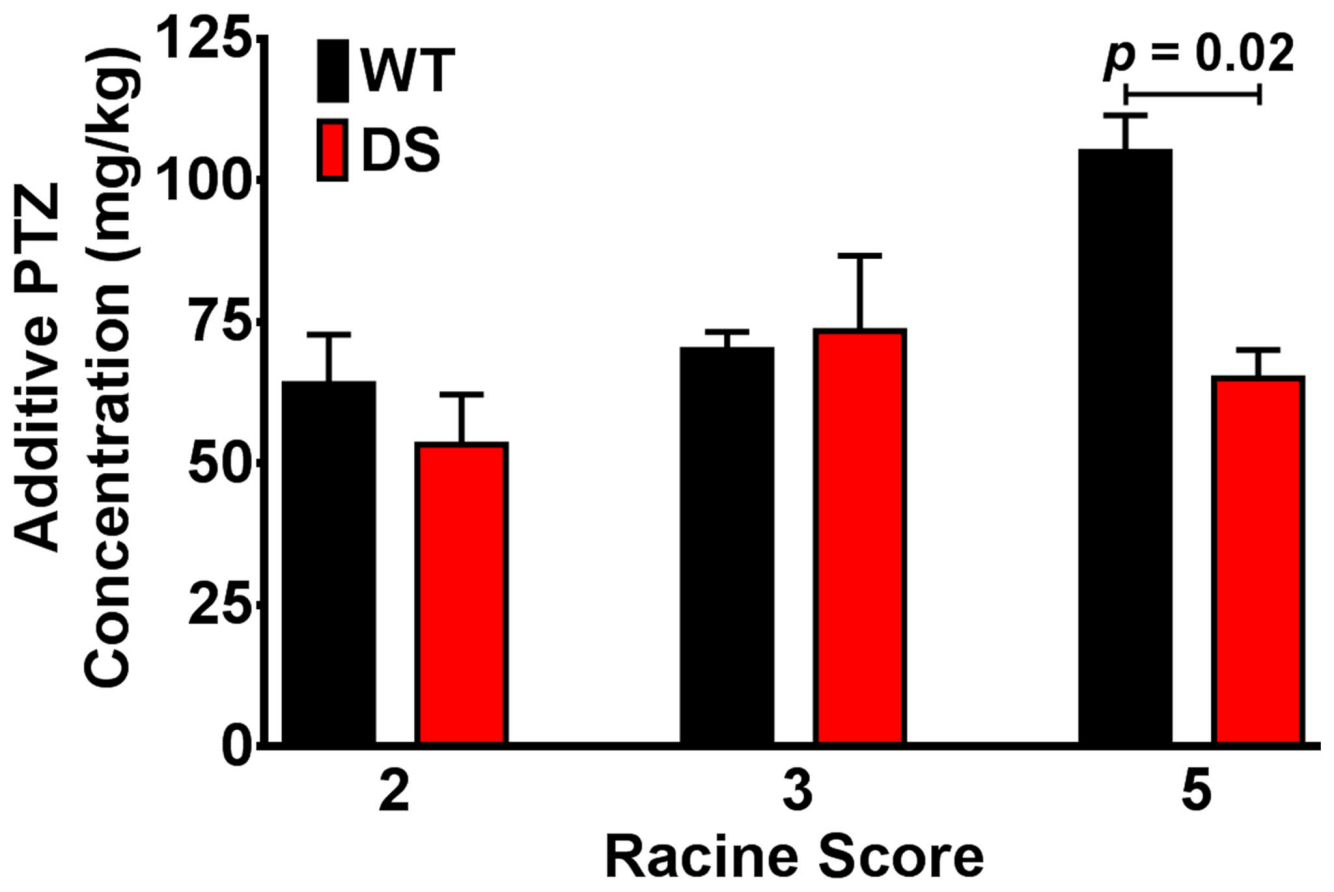
Decreased Threshold for PTZ Induced Seizures in DS Mice. WT and DS mice were administered incremental doses of pentylenetetrazole (PTZ), monitored for observable seizures, and classified on the Racine Scale.

We observed that 21% of DS mice die by P150, with 38% of these deaths occurring before P25 (similar to [25]), and 69% of the deaths by P52 (n = 75 for each group, Figure **5A**) [26]. A similar incidence of SUDEP was observed in DS mice that were implanted with radiotelemetry devices (Figure **5B**). At the termination of the *in vivo* radiotelemetry ECG study a subset of WT and DS mice were tested for the susceptibility to PTZ-induced convulsive seizures (rated on the Racine Scale). Administration of PTZ led to marked bradycardia, which may be similar to previously observed seizure-induced bradycardia [44] and similar to that observed in *Scn1a* ^*+/-*^ mice following acute hyperthermia induced seizures [25]. Interestingly, the initial injection of PTZ resulted in a sudden 32 ± 5% decrease in the HR, which was further diminished with increasing doses of PTZ (58 ± 3% of pre-drug). Consistent with the increased susceptibility to seizures in DS [26,45-48], and the previously documented incidence of spontaneous seizures in DS mice [26], the minimum additive concentration of PTZ after which a convulsive seizure was first observed was less in DS than in WT mice (*p* = 0.02 at Racine Scale 5, Figure **6**).

### In vivo ECG recordings suggest a cardiac mechanism for SUDEP in DS

**Figure 7:**
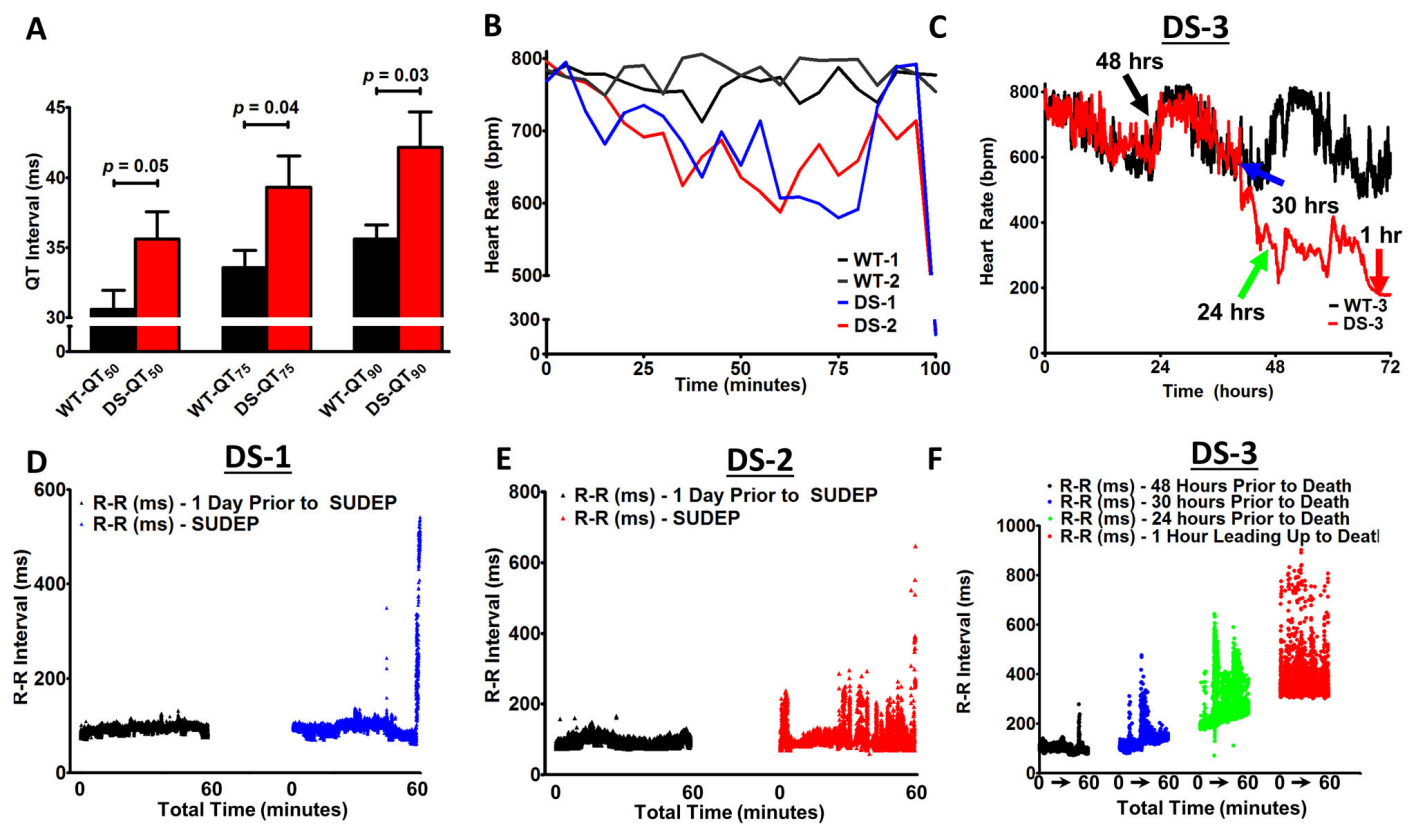
Altered Heart Rates Precede Death. A. DS mice exhibit significant QT prolongation (50 - 90%). B. Heart rates in DS mice decrease 100 min before death, followed by a sharp increase just prior to the terminal event, while the WT heart rates remains high and constant. (100 minutes = 10:16 PM in WT-1 and DS-1; 7:46 PM in WT-2 and DS-2). C. WT-3 and DS-3 HR cycling, followed by DS exhibiting sudden drops in heart rate in the 72 h preceding death. D and E. Increased R-R variability 60 min prior to SUDEP in DS-1 (blue) and DS-2 (red), respectively, with further increased variability immediately preceding the lethal arrhythmia, while 1 day prior at the same time the R-R interval was constant (black). F. Progressive bradycardia and increased R-R variability in DS-3 at several time points preceding an agonal state and euthanasia (denoted by colored arrows in C).

Consistent with the increased persistent I_Na_ and AP changes reported above, DS mice exhibited significant alterations in the ECG that may provide insights into a potential human DS cardiac phenotype. While there were no differences in HR, PQ interval, PR interval, or QRS duration, we observed a significant prolongation of the QT interval (HR corrected and uncorrected QT_50-90%_, Table **2** and Figure **7A**). We were able to record the events preceding spontaneous SUDEP (or near-SUDEP) in 3 of the 13 DS mice at P41, P45, and P51 (refered to as DS-1, DS-2, and DS-3), respectively. All 8 WT mice were alive at the end of the study (Figure **5B**). DS-1 and DS-2 died suddenly subsequent to ventricular fibrillation (VF), while DS-3 became moribund, with lack of movement, severe bradycardia, and hypothermia, and was euthanized for ethical reasons. All 3 DS mice that died exhibited R-R variability. Focal and idioventricular rhythms, conduction abnormalities, and ultimately a catastrophic cardiac arrhythmia preceded death in DS-1 and DS-2. One hundred minutes prior to putative SUDEP, which occurred at 7:46 PM and 10:16 PM in DS-1 and DS-2, respectively, the animals became bradycardic, followed by a sharp increase in the HR just prior to the terminal event (Figure **7B**). In contrast, the HR was steady in time matched WT-1 and WT-2 mice. DS-3 exhibited sudden non-lethal bradycardic events, resulting in large HR fluctations and progressive HR slowing (Figure **7C**). To further investigate the DS ECG phenotype preceding putative SUDEP, we analyzed the R-R interval. One day prior to death in DS-1 and DS-2 the R-R interval was regular. In contrast, 1 hour preceding death in both animals the R-R interval became irregular, with more frequent episodes of R-R variability preceding the fatal cardiac arrhythmia and sudden death (Figure **7D and E**). DS-3 developed increased R-R variability in the 48 hours preceding euthanasia, with the most pronounced R-R variability in the hour preceding death (Figure **7F**). 

**Table 2:** Telemetry ECG Measurements of various parameters illustrating a significant prolongation of the QT interval and the corrected QT interval (QT_c_). QT_c_ calculated by the Bazett formula.

	**Mouse**		
**Parameter**	**WT**	**DS**	**Significance**
**N = # mice**	N = 5	N = 13	
**RR**	87.3 ± 2.9	86.9 ± 2.2	*p* = 0.93
**PQ**	29.1 ± 1.3	28.0 ± 0.6	*p* = 0.46
**PR**	33.2 ± 0.9	32.4 ± 0.4	*p* = 0.41
**QRS**	7.1 ± 0.5	7.5 ± 0.3	*p* = 0.53
**QT_50_**	30.6 ± 1.4	35.6 ± 1.9	*p* = 0.05
**QT_75_**	33.6 ± 1.2	39.3 ± 2.2	*p* = 0.04
**QT_90_**	35.6 ± 1.0	42.2± 2.5	*p* = 0.03
**QT_50c_**	3.6 ± 0.2	4.2 ± 0.2	*p* = 0.04
**QT_75c_**	3.3 ± 0.2	3.8 ± 0.2	*p* = 0.06
**QT_90c_**	3.8 ± 0.2	4.5 ± 0.2	*p* = 0.03

Significant differences between the WT vs. DS myocytes are defined as *p* ≤ 0.05.

**Figure 8:**
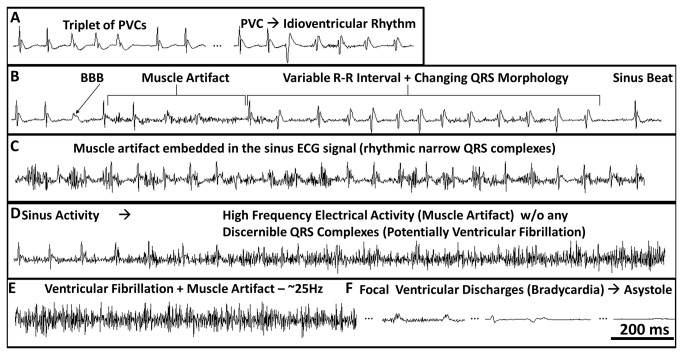
Cardiac Arrhythmias Precede SUDEP in DS. Lead II ECG traces illustrating cardiac arrhythmias preceding death. A-C. In mouse DS-2, muscle artifact consistent with convulsive seizures was preceded by idioventricular rhythms, including premature ventricular complexes (PVCs), bundle branch block (BBB), altered QRS morphology, and R-R variability. D and E. Initiation of high frequency electrical activity without any discernible sinus activity, consistent with VF. F. Low amplitude wide complex focal bradycardia with a BBB morphology, and eventual asystole.

**Figure 9:**
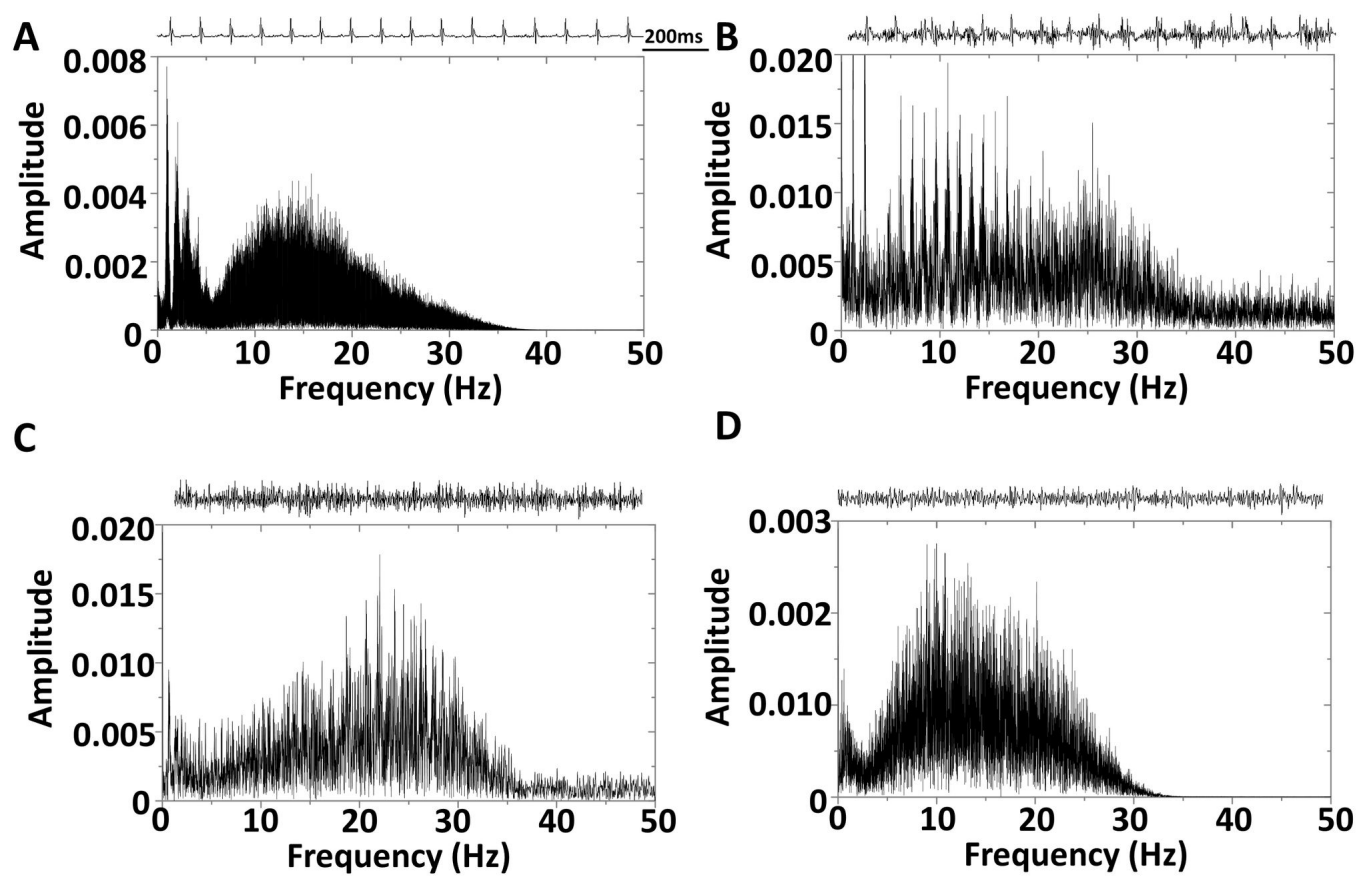
Dominant Frequency Analysis. *A. Sinus* rhythm 1 day prior to SUDEP, which is consistent with heart rate (728 bpm) analysis. B. Muscle artifact embedded in the sinus ECG (same as Figure 9 C) without any clear frequency peaks. C. High frequency electrical activity without any discernible sinus activity, consistent with VF (~25 Hz, same as Figure 9, D and E). D. PTZ induced seizures lead to a lower frequency electrical signal (~10 - 20 Hz). *Inset*: Representative snapshots of the ECG signal included in the fast-fourier transformation.

Cardiac arrhythmias in DS mice often preceded apparent spontaneous convulsive seizures observed as high frequency muscle artifacts (Figure **8**). These artifacts were not observed in untreated WT mice, but arose during PTZ-induced convulsive seizures in both WT and DS mice (Figures **9 D**). DS-1 and DS-2 exhibited periods of premature ventricular complexes (PVCs) and bundle branch block (BBB) that often preceded or occurred during apparent convulsive seizures (Figures **8** **, A and B**). In contrast, the incidence of PVCs and BBB during the PTZ study was low (3 of 8 mice with 1-3 PVCs per mouse over a 3 hour period), was not correlated with PTZ induced convulsive seizures, and was not closely coupled to the catastrophic event (occuring more than 30-60 minutes prior to death from status epilepticus). 

All 3 DS mice developed large R-R variability and idioventricular arrhythmias with changing QRS morphology, which were closely coupled with high frequency electrical activity, consistent with the signal during an apparent convulsive seizure (Figure **8** **, A-C**). Other than a few PVCs in WT-2, time matched ECG recordings from WT-1, WT-2, and WT-3 did not exhibit any of these ECG manifestations and arrhythmias that preceded sudden death in DS mice. In contrast to the ECG changes preceding death in DS-1 and DS-2, the R-R and QRS changes recorded after PTZ administration did not directly precede the convulsive seizures. Ultimately, DS-1 and DS-2 underwent a catastrophic event with high frequency electrical activity without any discernible QRS complexes. A contribution from muscle artifact cannot be entirely excluded, but the ECGs from both mice are consistent with VF (Figure **8, D and E**), as discussed below. 

Figure **9A** illustrates sinus activity with the corresponding Fast Fourier Transform (FFT) spectrum showing a dominant frequency peak at ~12 Hz that is consistent with the measured HR of 728 bpm. Figure **9B** is the FFT spectrum of an ECG pattern consistent with a convulsive seizure with the maintenance of sinus activity. There were not any clear dominant frequency peaks (ECG signal from Figure **8C**). Figure **9C** shows the FFT spectrum during the apparent VF ECG pattern. The dominant frequency of the complex electrical activity was ~25 Hz, which is consistent with previously reported frequencies of mouse VF [49,50]. To confirm that the muscle artifact from a spontaneous lethal convulsive seizure would not yield a similar FFT spectrum as in Figure **9C**, we assessed the FFT spectrum during a PTZ-induced convulsive seizure (Racine Scale 5, Figure **9D**). Unlike the dominant frequency peak at ~25 Hz in Figure **9C**, the ECG signal during a PTZ induced seizure was ~10 Hz (Figure **9D**). In summary, neither putative spontaneous seizures nor PTZ-induced seizures in DS mice, with and without identifiable QRS complexes embedded within the high frequency muscle artifact, respectively, yielded a peak at 25 Hz on the ECG FFT spectrum (Figure **9B and D**). Therefore, PTZ-induced seizures and death did not phenocopy the cardiac ECG phenotype of putative SUDEP in DS-1 and DS-2. Figure **9** demonstrates the sensitivity of FFT analysis to isolate the multipe frequency components and provides further evidence that one mechanism for SUDEP in DS may be cardiac arrhythmia.

Ultimately, DS-1 and DS-2 developed wide complex, low amplitude, and bradycardic focal discharges with a BBB morphology that progressively decreased in rate to eventual asystole (Figure **8F**). In contrast to DS-1 and DS-2, DS-3 did not have the opportunity to undergo a lethal cardiac arrhythmia, as it was euthanized for ethical reasons. Yet, during the final hour of ECG recordings DS-3, but not WT-3, became bradycardic (<180 bpm) and exhibited abrupt changes in the QRS morphology and amplitude. Some aspects of the spontaneous bradycardia and periods of bradycardia followed by tachycardia, observed here, are similar to effects reported in the *Scn1a* ^*+/-*^ DS mouse model following acute hyperthermic seizures [25], although there are key differences, for example, the absence of atrioventricular nodal block and the initiation of VF in our model. Nevertheless, taken together, these data support the hypothesis that significant cardiac pathophysiological changes occur in DS mice. 

## Discussion

SUDEP is a catastrophic, multi-system failure that involves seizures, changes in autonomic tone, respiratory dysregulation, and cardiac arrhythmias [5,7,51]. Recent work suggested that parasympathetic hyperactivity following hyperthermia induced tonic-clonic seizures resulted in severe bradycardia and death in the *Scn1a* ^*+/-*^ mouse model of DS [25]. This study, however, left open important gaps in our knowledge: first, whether cardiac myocytes isolated from a mouse model of DS have altered excitability and second, whether DS mice exhibit cardiac dysfunction following spontaneous seizures. Here, we used a multilevel approach to investigate the DS cardiac phenotype in a global knockin mouse model expressing a human DS mutation. Electrophysiological recordings of acutely dissociated ventricular myocytes demonstrated that *Scn1a* haploinsufficiency leads to a 2-fold increase in transient and persistent I_Na_ density. The pharmacological and biophysical properties of I_Na_ in the DS myocytes suggest that the observed increase in I_Na_ is the result of an increased number of functional Na_v_1.5 channels at the plasma membrane. DS ventricular myocytes exhibited alterations in AP morphology and incidences of triggered activity. *In vivo*, DS mice developed spontaneous seizures and pathological ECG manifestations, including bradycardia, idioventricular rhythms, RR variability, PVCs, BBB, and VF. Ultimately, these results provide mechanistic insights into how alterations in cardiac electrical function establish ideal conditions for arrhythmogenesis and SUDEP. Thus, DS mutations in *Scn1a* lead not only to alterations in neuronal excitability, but also to cardiac electrophysiological abnormalities in isolated ventricular myocytes, contributing to the mechanism underlying SUDEP. 

### DS venticular myocytes have increased I_Na_ density with AP changes

Neuronal hyperexcitability in DS mouse models has been proposed to occur through a selective decrease in I_Na_ in inhibitory neurons [25,26,34,52]. Interestingly, more recent results demonstrate that at P21-24, an age older than previously investigated, I_Na_ is increased in excitatory pyramidal neurons in Scn1a^+/-^ mice [53]. Further, DS patient-specific forebrain-like neurons generated from iPSCs have increased I_Na_ in both excitatory and inhibitory neuronal cell types [32]. Here, we observed a significant increase in TTX-R I_Na_ density and hyperpolarizing shifts in the voltage dependence of I_Na_ conductance and availability in DS cardiac myocytes. Our data are consistent with previous reports showing that increased Na_v_1.5 expression leads to hyperpolarizing shifts in the voltage dependence of I_Na_ conductance and availability [28,29]. These results suggest that the increased I_Na_ in DS can be explained by increased functional TTX-R Na_v_1.5 activity. In another mouse model of DS (Scn1b null mice [54]) there was a 2-fold increase in Na_v_1.5-mediated transient and persistent I_Na_. Furthermore, in Scn1b null mice and in ventricular myocytes over-expressing human SCN5A [28] there was action potential prolongation, with prolonged QT and RR intervals in the Scn1b mice [54]. Increased transient I_Na_ is predicted to provide more depolarizing current during the AP upstroke, resulting in a lower threshold of current injection to elicit an AP, and increased AP upstroke velocity. Increased transient I_Na_ alone would increase the safety factor and preserve the stability of impulse propagation [18]. However, as observed in models of LQTS-3 and Na_v_1.5 overexpression, increased persistent I_Na_ disrupts the balance between depolarizing and repolarizing currents, leading to APD/QT prolongation, EADs, arrhythmias, and sudden cardiac death [28,55]. 

### DS Mice Exhibit LQTS Phenotype

Similar to the I_Na_ recordings from DS mice presented here, our previous work using the Scn1b null mouse model of DS demonstrated proportional increases in transient and persistent I_Na_ [54]. In contrast, genetic and pharmacological models of increased persistent I_Na_ leading to LQTS3 exhibit a disproportionate increase in persistent I_Na_, with little change in transient I_Na_ [55-58]. As we previously demonstrated however [28], regardless of whether the increase in persistent I_Na_ scales with the transient current, an absolute increase in persistent I_Na_ disturbs the balance of inward and outward currents during the AP plateau (a time of high membrane resistance). Subsequently, this leads to APD prolongation and EAD formation, which is a known trigger for the initiation of arrhythmias and sudden cardiac death [19,59]. 

It remains incompletely understood whether the QT interval is prolonged in DS patients. QT prolongation has been shown to be a predisposing interictal and peri-ictal factor that ultimately leads to SUDEP in some (non-DS) epilepsy patients [7]. QT prolongation has been observed in children and adult patients with chronic epilepsy, during epileptic discharges, and the QT prolongation is even more prevalent in SUDEP vs. without SUDEP epileptic patients [4,60-64]. Interestingly, 33-44% of epilepsy patients and mouse models of epilepsy are prone to cardiac arrhythmias and LQTS, and conversely, one-third of LQTS patients have a history of seizures [8,65,66]. In two studies that examined ECG parameters in patients with DS, increased QT dispersion [67] and decreased heart rate variability [67,68] were observed, with no changes in the QT interval [67,68]. Studies that have examined the QT interval in DS mouse models have yielded contrasting results, with QT prolongation observed in the Scn1b null model and no change in the Scn1a^+/-^ model [25,54]. The present study, using a human SCN1A knockin mutant mouse model, is the first to record ECG properties in conscious, unrestrained, and telemetered DS mice that have regained circadian heart rate, temperature, and activity cycling (e.g., Figure 7C). 

### DS mutations lead to alterations in the expression of other ion channels

Changes in the expression of a single ion channel gene often result in changes in the expression levels of other ion channel genes [29,69,70]. *Scn1a* null mice have increased Na**_*v*_**1.3 expression in the hippocampus [34]. *Scn1b* null mice have decreased Na**_*v*_**1.1 and increased Na**_*v*_**1.3 expression in the hippocampus, increased *Scn5a*/Na**_*v*_**1.5 expression and ^3^H-saxitoxin binding (suggesting increased TTX-S VGSC expression) in the heart [54], as well as altered TTX-R and TTX-S I_Na_ biophysical properties and decreased Na**_*v*_**1.9 expression in dorsal-root-ganglia (DRG) [48,69]. DS patient-specific iPSC excitatory and inhibitory neurons have increased I_Na_ in spite of *SCN1A* haploinsufficiency [32]. These compensatory changes in ion channel expression are not limited to VGSCs, as there is also a reduction in K^+^ current in *Scn1b* null DRGs and cortical pyramidal neurons, contributing to hyperexcitability [69,71]. Dissociated DRG neurons from *Scn1b* null mice have a reduction in not only I_Na_ density, but also in K_*v*_4.2 expression and I_A_ density [74]. Furthermore, Na_v_β1 has been shown to interact with K_*v*_4.2 [71]. Cortical neurons from *Scn1b* null mice exhibited reduced I_A_ current, and cortical pyramidal neurons exhibited APD prolongation with repetitive firing [71]. Additionally, Na**_*v*_**1.5 and Kir2.1 have been shown to be part of a macromolecular complex, with reciprocal interactions between Na_v_1.5 and Kir2.1 expression, and subsequent changes in I_Na_ and I_K1_ density [29]. Here, we show that *Scn1a* haploinsufficiency leads to increased functional expression of TTX-R I_Na_ in the heart. Taken together, these data suggest a homeostatic-like mechanism in response to VGSC subunit gene mutation that overcompensates by increased activity of a different VGSC subunit. 

It is not surprising that we were unable to detect a measureable decrease in TTX-S I_Na_ in our experiments. Na_v_1.1 is one of at least three known TTX-S VGSCs expressed in cardiac myocytes, and TTX-S I_Na_ contributes only ~10% of the global I_Na_ [11,14,17,42,43]. Thus, it is unlikely that small decreases in TTX-S I_Na_, due to *Scn1a* haploinsufficency, would be resolvable using the whole cell voltage clamp technique. In future studies, the use of super-resolution imaging, as in [72], will allow positioning of the recording electrode directly at the T-tubules and in the triad, where TTX-S VGSCs are localized, thus enabling more precise measurement of small, localized changes in cardiac TTX-S I_Na_. In spite of this, it is possible that decreases in Na_v_1.1 expression in the T-tubules may disturb the macromolecular complex that includes the Na^+^-Ca^2+^ exchanger, Na+/K+-ATPase, inositol triphosphate receptor, Ca^2+^-calmodulin dependent protein kinase-II, and ankyrin-B [73,74]. Mutations in ankyrin-B are known to lead to altered Ca^2+^ handling and pathological ECG changes, including QT prolongation, bradycardia, sinus arrhythmia, idiopathic ventricular fibrillation, catecholaminergic polymorphic ventricular tachycardia, and risk of sudden death [75,76]. These results show that microdomain changes at the T-tubules can lead to whole cell implications. Here, in our DS model, altered Ca^2+^ handling downstream of reductions in Na_v_1.1 expression at the T-tubules may indirectly affect Na_v_1.5 function via changes in the binding of calmodulin to the channel or altered Ca^2+^-calmodulin dependent protein kinase-II-mediated channel phosphorylation, ultimately resulting in increased I_Na_ and arrhythmia [77,78]. Testing this hypothesis will be the focus of future studies.

### Cardiac arrhythmias provide a mechanism for SUDEP in DS

Ion channelopathies are multi-organ diseases. Simultaneous electroencephalogram (EEG) and ECG studies confirm the high prevalence (33-44%) of arrhythmias in epileptic patients [8]. Conversely, approximatly one-third of LQTS patients have a history of seizures [65]. We propose that SUDEP may be caused by mutations in ion channel genes that are expressed in both the brain and the heart (e.g., *KCNQ1* [8], *KCNH2* [65], *SCN1B* [54], *SCN5A* [79], *SCN8A* [15], and *SCN1A* [this study and [25]). Just prior to death, some SUDEP patients [5] and LQTS mutant mice [8] exhibit loss of EEG activity, cardiorespiratory changes, and ultimately fatal cardiac arrhythmias. Sudden death due to cardiac arrhythmias in the mice shown here is consistent with published factors that precipitated and accompanied SUDEP [7]. Our mouse data mirror a human case of near-SUDEP in which the patient developed VF following a generalized seizure and ventricular tachycardia [66]. This study is the first to reveal the implications of DS mutations in *Scn1a* on the incidence and mechanisms of arrhythmias and SUDEP due to changes in cardiac myocyte excitability, and suggest targets for risk assessment and intervention to prevent SUDEP in DS and perhaps other epileptic channelopathies.

## References

[1] Escayg A, Goldin AL (2010) Sodium channel SCN1A and epilepsy: mutations and mechanisms. Epilepsia 51: 1650-1658. doi:10.1111/j.1528-1167.2010.02640.x. PubMed: 20831750. 20831750 PMC2937162

[2] Dravet C, Bureau M, Oguni H, Fukuyama Y, Cokar O (2005) Severe myoclonic epilepsy in infancy: Dravet syndrome. Adv_Neurol 95: 71-102. PubMed: 15508915. 15508915

[3] Oakley JC, Kalume F, Catterall WA (2011) Insights into pathophysiology and therapy from a mouse model of Dravet syndrome. Epilepsia 52 Suppl 2: 59-61. doi:10.1111/j.1528-1167.2011.03004.x. PubMed: 21463282. 21463282 PMC3547637

[4] Surges R, Adjei P, Kallis C, Erhuero J, Scott CA et al. (2010) Pathologic cardiac repolarization in pharmacoresistant epilepsy and its potential role in sudden unexpected death in epilepsy: a case-control study. Epilepsia 51: 233-242. doi:10.1111/j.1528-1167.2009.02330.x. PubMed: 19817816. 19817816

[5] Shorvon S, Tomson T (2011) Sudden unexpected death in epilepsy. Lancet, 378: 2028–38. PubMed: 21737136. 21737136 10.1016/S0140-6736(11)60176-1

[6] Schuele SU, Widdess-Walsh P, Bermeo A, Lüders HO (2007) Sudden unexplained death in epilepsy: the role of the heart. Cleve_Clin_J_Med 74 Suppl 1: S121-S127. PubMed: 17455560. 17455560 10.3949/ccjm.74.suppl_1.s121

[7] Surges R, Thijs RD, Tan HL, Sander JW (2009) Sudden unexpected death in epilepsy: risk factors and potential pathomechanisms. NatRev. Neurologist 5: 492-504. 10.1038/nrneurol.2009.118 19668244

[8] Goldman AM, Glasscock E, Yoo J, Chen TT, Klassen TL et al. (2009) Arrhythmia in heart and brain: KCNQ1 mutations link epilepsy and sudden unexplained death. Sci Transl_Med 1: 2ra6 PubMed: 20368164. 10.1126/scitranslmed.3000289 PMC2951754 20368164

[9] Claes L, Del-Favero J, Ceulemans B, Lagae L, Van Broeckhoven C et al. (2001) De novo mutations in the sodium-channel gene SCN1A cause severe myoclonic epilepsy of infancy. Am_J_Hum_Genet 68: 1327-1332. PubMed: 11359211. 11359211 10.1086/320609 PMC1226119

[10] Meisler MH, Kearney JA (2005) Sodium channel mutations in epilepsy and other neurological disorders. J_Clin_Invest 115: 2010-2017. PubMed: 16075041. 16075041 10.1172/JCI25466 PMC1180547

[11] Maier SK, Westenbroek RE, McCormick KA, Curtis R, Scheuer T et al. (2004) Distinct subcellular localization of different sodium channel alpha and beta subunits in single ventricular myocytes from mouse heart. Circulation 109: 1421-1427. doi:10.1161/01.CIR.0000121421.61896.24. PubMed: 15007009. 15007009

[12] Maier SK, Westenbroek RE, Yamanushi TT, Dobrzynski H, Boyett MR et al. (2003) An unexpected requirement for brain-type sodium channels for control of heart rate in the mouse sinoatrial node. Proc_Natl_Acad Sci U_S_A 100: 3507-3512. PubMed: 12631690. 12631690 10.1073/pnas.2627986100 PMC152323

[13] Inoue N, Ohkusa T, Nao T, Lee JK, Matsumoto T et al. (2004) Rapid electrical stimulation of contraction modulates gap junction protein in neonatal rat cultured cardiomyocytes: involvement of mitogen-activated protein kinases and effects of angiotensin II-receptor antagonist. Jamcollcardiol 44: 914-922. PubMed: 15312880. 10.1016/j.jacc.2004.05.054 15312880

[14] Dhar Malhotra J, Chen C, Rivolta I, Abriel H, Malhotra R et al. (2001) Characterization of sodium channel alpha- and beta-subunits in rat and mouse cardiac myocytes. Circulation 103: 1303-1310. doi:10.1161/01.CIR.103.9.1303. PubMed: 11238277. 11238277

[15] Noujaim SF, Kaur K, Milstein M, Jones JM, Furspan P et al. (2012) A null mutation of the neuronal sodium channel NaV1.6 disrupts action potential propagation and excitation-contraction coupling in the mouse heart. FASEB J 26: 63-72. doi:10.1096/fj.10-179770. PubMed: 21948246. 21948246 PMC3250234

[16] Baruscotti M, Westenbroek R, Catterall WA, DiFrancesco D, Robinson RB (1997) The newborn rabbit sino-atrial node expresses a neuronal type I-like Na+ channel. J Physiol 498 ( 3): 641-648. PubMed: 9051576. 9051576 10.1113/jphysiol.1997.sp021889 PMC1159181

[17] Kaufmann SG, Westenbroek RE, Maass AH, Lange V, Renner A et al. (2013) Distribution and function of sodium channel subtypes in human atrial myocardium. J Mol Cell Cardiol, 61: 133–41. PubMed: 23702286. 23702286 10.1016/j.yjmcc.2013.05.006 PMC3906922

[18] Kléber AG, Rudy Y (2004) Basic mechanisms of cardiac impulse propagation and associated arrhythmias. Physiol Rev 84: 431-488. doi:10.1152/physrev.00025.2003. PubMed: 15044680. 15044680

[19] Zareba W, Cygankiewicz I (2008) Long QT syndrome and short QT syndrome. Prog_Cardiovasc_Dis 51: 264-278. PubMed: 19026859. 19026859 10.1016/j.pcad.2008.10.006

[20] George AL Jr. (2005) Inherited disorders of voltage-gated sodium channels. J_Clin_Invest 115: 1990-1999. PubMed: 16075039. 16075039 10.1172/JCI25505 PMC1180550

[21] Catterall WA, Kalume F, Oakley JC (2010) NaV1.1 channels and epilepsy. JPhysiol J Physiol 588: 1849-1859. PubMed: 20194124. 20194124 10.1113/jphysiol.2010.187484 PMC2901973

[22] Trudeau MM, Dalton JC, Day JW, Ranum LP, Meisler MH (2006) Heterozygosity for a protein truncation mutation of sodium channel SCN8A in a patient with cerebellar atrophy, ataxia, and mental retardation. Med_Genet 43: 527-530. 10.1136/jmg.2005.035667 PMC2564538 16236810

[23] Papadatos GA, Wallerstein PM, Head CE, Ratcliff R, Brady PA et al. (2002) Slowed conduction and ventricular tachycardia after targeted disruption of the cardiac sodium channel gene Scn5a. Proc Natl_Acad_Sci_U_S_A 99: 6210-6215. PubMed: 11972032. 11972032 10.1073/pnas.082121299 PMC122928

[24] Lei M, Jones SA, Liu J, Lancaster MK, Fung SS et al. (2004) Requirement of neuronal- and cardiac-type sodium channels for murine sinoatrial node pacemaking. JPhysiol 559: 835-848 10.1113/jphysiol.2004.068643 PMC1665172 15254155

[25] Kalume F, Westenbroek RE, Cheah CS, Yu FH, Oakley JC et al. (2013) Sudden unexpected death in a mouse model of Dravet syndrome. J Clin Invest 123: 1798-1808. doi:10.1172/JCI66220. PubMed: 23524966. 23524966 PMC3613924

[26] Ogiwara I, Miyamoto H, Morita N, Atapour N, Mazaki E et al. (2007) Nav1.1 localizes to axons of parvalbumin-positive inhibitory interneurons: a circuit basis for epileptic seizures in mice carrying an Scn1a gene mutation. JNeurosci J Neurosci 27: 5903-5914. PubMed: 17537961. 17537961 10.1523/JNEUROSCI.5270-06.2007 PMC6672241

[27] Cerrone M, Noujaim SF, Tolkacheva EG, Talkachou A, O'Connell R et al. (2007) Arrhythmogenic mechanisms in a mouse model of catecholaminergic polymorphic ventricular tachycardia. Circ_Res 101: 1039-1048. PubMed: 17872467. 17872467 10.1161/CIRCRESAHA.107.148064 PMC2515360

[28] Auerbach DS, Grzeda KR, Furspan PB, Sato PY, Mironov S et al. (2011) Structural heterogeneity promotes triggered activity, reflection and arrhythmogenesis in cardiomyocyte monolayers. JPhysiol 589: 2363-2381 10.1113/jphysiol.2010.200576 PMC3098708 21486795

[29] Milstein ML, Musa H, Balbuena DP, Anumonwo JM, Auerbach DS et al. (2012) Dynamic reciprocity of sodium and potassium channel expression in a macromolecular complex controls cardiac excitability and arrhythmia. Proc Natl Acad Sci U S A 109: E2134-E2143. doi:10.1073/pnas.1109370109. PubMed: 22509027. 22509027 PMC3412015

[30] Brackenbury WJ, Davis TH, Chen C, Slat EA, Detrow MJ et al. (2008) Voltage-gated Na+ channel beta1 subunit-mediated neurite outgrowth requires Fyn kinase and contributes to postnatal CNS development in vivo. J Neurosci 28: 3246-3256. doi:10.1523/JNEUROSCI.5446-07.2008. PubMed: 18354028. 18354028 PMC6670697

[31] Lowe JS, Palygin O, Bhasin N, Hund TJ, Boyden PA et al. (2008) Voltage-gated Nav channel targeting in the heart requires an ankyrin-G dependent cellular pathway. J Cell Biol 180: 173-186. doi:10.1083/jcb.200710107. PubMed: 18180363. 18180363 PMC2213608

[32] Liu Y, Lopez-Santiago LF, Yuan Y, Jones JM, Zhang H et al. (2013) Dravet syndrome patient-derived neurons suggest a novel epilepsy mechanism. Ann Neurol, 74: 128–39. PubMed: 23821540. 23821540 10.1002/ana.23897 PMC3775921

[33] Chen Y, Yu FH, Sharp EM, Beacham D, Scheuer T et al. (2008) Functional properties and differential neuromodulation of Na(v)1.6 channels. Mol_Cell Neurosci 38: 607-615. doi:10.1016/j.mcn.2008.05.009. PubMed: 18599309. 18599309 PMC3433175

[34] Yu FH, Mantegazza M, Westenbroek RE, Robbins CA, Kalume F et al. (2006) Reduced sodium current in GABAergic interneurons in a mouse model of severe myoclonic epilepsy in infancy. Nat_Neurosci 9: 1142-1149. PubMed: 16921370. 16921370 10.1038/nn1754

[35] Yamaji N, Little MJ, Nishio H, Billen B, Villegas E et al. (2009) Synthesis, solution structure, and phylum selectivity of a spider delta-toxin that slows inactivation of specific voltage-gated sodium channel subtypes. Biol_Chem 284: 24568-24582. PubMed: 19592486. 10.1074/jbc.M109.030841 PMC2782047 19592486

[36] Petitprez S, Zmoos AF, Ogrodnik J, Balse E, Raad N et al. (2011) SAP97 and dystrophin macromolecular complexes determine two pools of cardiac sodium channels Nav1.5 in cardiomyocytes. Circ_Res 108: 294-304. PubMed: 21164104. 21164104 10.1161/CIRCRESAHA.110.228312

[37] Fletcher EV, Kullmann DM, Schorge S (2011) Alternative splicing modulates inactivation of type 1 voltage-gated sodium channels by toggling an amino acid in the first S3-S4 linker. Biol_Chem 286: 36700-36708. PubMed: 21890636. 10.1074/jbc.M111.250225 PMC3196094 21890636

[38] Patino GA, Brackenbury WJ, Bao Y, Lopez-Santiago LF, O'Malley HA et al. (2011) Voltage-Gated Na+ Channel {beta}1B: A Secreted Cell Adhesion Molecule Involved in Human Epilepsy. JNeurosci 31: 14577-14591 10.1523/JNEUROSCI.0361-11.2011 PMC3212034 21994374

[39] Estrada G, Restano-Cassulini R, Ortiz E, Possani LD, Corzo G (2011) Addition of positive charges at the C-terminal peptide region of CssII, a mammalian scorpion peptide toxin, improves its affinity for sodium channels Nav1.6. Peptides 32: 75-79. doi:10.1016/j.peptides.2010.11.001. PubMed: 21078353. 21078353

[40] Chatelier A, Zhao J, Bois P, Chahine M (2010) Biophysical characterisation of the persistent sodium current of the Nav1.6 neuronal sodium channel: a single-channel analysis. Pflugers Arch 460: 77-86. doi:10.1007/s00424-010-0801-9. PubMed: 20204400. 20204400

[41] Mercier A, Clément R, Harnois T, Bourmeyster N, Faivre JF et al. (2012) The beta1-subunit of Na(v)1.5 cardiac sodium channel is required for a dominant negative effect through alpha-alpha interaction. PLOS ONE 7: e48690. doi:10.1371/journal.pone.0048690. PubMed: 23133651. 23133651 PMC3486797

[42] Brette F, Orchard CH (2006) Density and sub-cellular distribution of cardiac and neuronal sodium channel isoforms in rat ventricular myocytes. Biochem_Biophys_Res_Commun 348: 1163-1166. PubMed: 16904633. 16904633 10.1016/j.bbrc.2006.07.189

[43] Haufe V, Cordeiro JM, Zimmer T, Wu YS, Schiccitano S et al. (2005) Contribution of neuronal sodium channels to the cardiac fast sodium current INa is greater in dog heart Purkinje fibers than in ventricles. Cardiovasc_Res 65: 117-127. PubMed: 15621039. 15621039 10.1016/j.cardiores.2004.08.017

[44] Nashef L, Hindocha N, Makoff A (2007) Risk factors in sudden death in epilepsy (SUDEP): the quest for mechanisms. Epilepsia 48: 859-871. doi:10.1111/j.1528-1167.2007.01082.x. PubMed: 17433051. 17433051

[45] Dravet C, Bureau M, Dalla BB, Guerrini R (2011) Severe myoclonic epilepsy in infancy (Dravet syndrome) 30 years later. Epilepsia 52 Suppl 2: 1-2. doi:10.1111/j.1528-1167.2010.02921.x. PubMed: 21463271. 21463271

[46] Guerrini R, Aicardi J (2003) Epileptic encephalopathies with myoclonic seizures in infants and children (severe myoclonic epilepsy and myoclonic-astatic epilepsy). J_Clin_Neurophysiol 20: 449-461. PubMed: 14734934. 14734934 10.1097/00004691-200311000-00007

[47] Oakley JC, Kalume F, Yu FH, Scheuer T, Catterall WA (2009) Temperature- and age-dependent seizures in a mouse model of severe myoclonic epilepsy in infancy. Proc_Natl_Acad Sci U_S_A 106: 3994-3999. PubMed: 19234123. 19234123 10.1073/pnas.0813330106 PMC2656193

[48] Chen C, Westenbroek RE, Xu X, Edwards CA, Sorenson DR et al. (2004) Mice lacking sodium channel beta1 subunits display defects in neuronal excitability, sodium channel expression, and nodal architecture. JNeurosci 24: 4030-4042 10.1523/JNEUROSCI.4139-03.2004 PMC6729427 15102918

[49] Noujaim SF, Pandit SV, Berenfeld O, Vikstrom K, Cerrone M et al. (2007) Up-regulation of the inward rectifier K+ current (I K1) in the mouse heart accelerates and stabilizes rotors. JPhysiol 578: 315-326 10.1113/jphysiol.2006.121475 PMC2075137 17095564

[50] Noujaim SF, Berenfeld O, Kalifa J, Cerrone M, Nanthakumar K et al. (2007) Universal scaling law of electrical turbulence in the mammalian heart. Proc Natl Acad Sci U S A 104: 20985-20989. doi:10.1073/pnas.0709758104. PubMed: 18093948. 18093948 PMC2409253

[51] Nei M, Hays R (2010) Sudden unexpected death in epilepsy. Curr Neurol Neurosci Rep 10: 319-326. doi:10.1007/s11910-010-0116-4. PubMed: 20446062. 20446062

[52] Cheah CS, Yu FH, Westenbroek RE, Kalume FK, Oakley JC et al. (2012) Specific deletion of NaV1.1 sodium channels in inhibitory interneurons causes seizures and premature death in a mouse model of Dravet syndrome. Proc Natl Acad Sci U S A 109: 14646-14651. doi:10.1073/pnas.1211591109. PubMed: 22908258. 22908258 PMC3437823

[53] Mistry A, Miller A, Thompson C, Kearney J, George AL (2012) Strain and age-dependent differences in hippocampal neuron sodium current densities in a mouse model of Dravet syndrome. New Orleans, LA.: Society for Neuroscience.

[54] Lopez-Santiago LF, Meadows LS, Ernst SJ, Chen C, Malhotra JD et al. (2007) Sodium channel Scn1b null mice exhibit prolonged QT and RR intervals. Jmolcell Cardiol 43: 636-647. PubMed: 17884088. 10.1016/j.yjmcc.2007.07.062 PMC2099572 17884088

[55] Nuyens D, Stengl M, Dugarmaa S, Rossenbacker T, Compernolle V et al. (2001) Abrupt rate accelerations or premature beats cause life-threatening arrhythmias in mice with long-QT3 syndrome. Nat_Med 7: 1021-1027. PubMed: 11533705. 11533705 10.1038/nm0901-1021

[56] Terrenoire C, Wang K, Tung KW, Chung WK, Pass RH et al. (2013) Induced pluripotent stem cells used to reveal drug actions in a long QT syndrome family with complex genetics. J Gen Physiol 141: 61-72. PubMed: 23277474. 23277474 10.1085/jgp.201210899 PMC3536519

[57] Pignier C, Revenaz C, Rauly-Lestienne I, Cussac D, Delhon A et al. (2007) Direct protective effects of poly-unsaturated fatty acids, DHA and EPA, against activation of cardiac late sodium current: a mechanism for ischemia selectivity. Basic Res_Cardiol 102: 553-564. doi:10.1007/s00395-007-0676-x. PubMed: 17891522. 17891522

[58] Malan D, Friedrichs S, Fleischmann BK, Sasse P (2011) Cardiomyocytes obtained from induced pluripotent stem cells with long-QT syndrome 3 recapitulate typical disease-specific features in vitro. Circ Res 109: 841-847. doi:10.1161/CIRCRESAHA.111.243139. PubMed: 21799153. 21799153

[59] Moss AJ, Kass RS (2005) Long QT syndrome: from channels to cardiac arrhythmias. J_Clin_Invest 115: 2018-2024. PubMed: 16075042. 16075042 10.1172/JCI25537 PMC1180552

[60] Tavernor SJ, Brown SW, Tavernor RM, Gifford C (1996) Electrocardiograph QT lengthening associated with epileptiform EEG discharges--a role in sudden unexplained death in epilepsy? Seizure 5: 79-83. doi:10.1016/S1059-1311(96)80067-7. PubMed: 8777558. 8777558

[61] Oppenheimer S (1990) Cardiac dysfunction during seizures and the sudden epileptic death syndrome. Jrsocmed 83: 134-136. PubMed: 2182856. 10.1177/014107689008300302 PMC1292552 2182856

[62] Surges R, Taggart P, Sander JW, Walker MC (2010) Too long or too short? New insights into abnormal cardiac repolarization in people with chronic epilepsy and its potential role in sudden unexpected death. Epilepsia 51: 738-744. doi:10.1111/j.1528-1167.2010.02571.x. PubMed: 20384763. 20384763

[63] Kändler L, Fiedler A, Scheer K, Wild F, Frick U et al. (2005) Early post-convulsive prolongation of QT time in children. Acta Paediatr 94: 1243-1247. PubMed: 16278992. 16278992 10.1111/j.1651-2227.2005.tb02083.x

[64] Brotherstone R, Blackhall B, McLellan A (2010) Lengthening of corrected QT during epileptic seizures. Epilepsia 51: 221-232. doi:10.1111/j.1528-1167.2009.02281.x. PubMed: 19732135. 19732135

[65] Johnson JN, Hofman N, Haglund CM, Cascino GD, Wilde AA et al. (2009) Identification of a possible pathogenic link between congenital long QT syndrome and epilepsy. Neurology 72: 224-231. doi:10.1212/01.wnl.0000335760.02995.ca. PubMed: 19038855. 19038855 10.1212/01.wnl.0000335760.02995.ca PMC2677528

[66] Espinosa PS, Lee JW, Tedrow UB, Bromfield EB, Dworetzky BA (2009) Sudden unexpected near death in epilepsy: malignant arrhythmia from a partial seizure. Neurology 72: 1702-1703. doi:10.1212/WNL.0b013e3181a55f90. PubMed: 19433745. 19433745

[67] Ergul Y, Ekici B, Tatli B, Nisli K, Ozmen M (2013) QT and P wave dispersion and heart rate variability in patients with Dravet syndrome. Acta Neurol Belg 113: 161-166. doi:10.1007/s13760-012-0140-z. PubMed: 23065439. 23065439

[68] Delogu AB, Spinelli A, Battaglia D, Dravet C, De Nisco A et al. (2011) Electrical and autonomic cardiac function in patients with Dravet syndrome. Epilepsia 52 Suppl 2: 55-58. doi:10.1111/j.1528-1167.2011.03003.x. PubMed: 21463281. 21463281

[69] Lopez-Santiago LF, Brackenbury WJ, Chen C, Isom LL (2011) Na+ channel Scn1b gene regulates dorsal root ganglion nociceptor excitability in vivo. Biol_Chem 286: 22913-22923. PubMed: 21555511. 10.1074/jbc.M111.242370 PMC3123059 21555511

[70] Sato PY, Musa H, Coombs W, Guerrero-Serna G, Patiño GA et al. (2009) Loss of plakophilin-2 expression leads to decreased sodium current and slower conduction velocity in cultured cardiac myocytes. Circ_Res 105: 523-526. PubMed: 19661460. 19661460 10.1161/CIRCRESAHA.109.201418 PMC2742576

[71] Marionneau C, Carrasquillo Y, Norris AJ, Townsend RR, Isom LL et al. (2012) The sodium channel accessory subunit Navbeta1 regulates neuronal excitability through modulation of repolarizing voltage-gated K(+) channels. J Neurosci 32: 5716-5727. doi:10.1523/JNEUROSCI.6450-11.2012. PubMed: 22539834. 22539834 PMC3347704

[72] Agullo-Pascual E, Reid DA, Keegan S, Sidhu M, Fenyö D et al. (2013) Super-resolution fluorescence microscopy of the cardiac connexome reveals plakophilin-2 inside the connexin43 plaque. Cardiovasc Res. PubMed: 23929525 10.1093/cvr/cvt191 PMC3797628 23929525

[73] Mohler PJ, Davis JQ, Bennett V (2005) Ankyrin-B coordinates the Na/K ATPase, Na/Ca exchanger, and InsP3 receptor in a cardiac T-tubule/SR microdomain. PLOS Biol 3: e423. doi:10.1371/journal.pbio.0030423. PubMed: 16292983. 16292983 PMC1287507

[74] J O-Uchi, K Komukai, Y Kusakari, T Obata, K Hongo, et al. (2005) alpha1-adrenoceptor stimulation potentiates L-type Ca2+ current through Ca2+/calmodulin-dependent PK II (CaMKII) activation in rat ventricular myocytes. Proc Natl Acad Sci U S A 102: 9400-9405. doi:10.1073/pnas.0503569102. PubMed: 15964981. 10.1073/pnas.0503569102 PubMed: 15964981 15964981 PMC1166620

[75] Mohler PJ, Schott JJ, Gramolini AO, Dilly KW, Guatimosim S et al. (2003) Ankyrin-B mutation causes type 4 long-QT cardiac arrhythmia and sudden cardiac death. Nature 421: 634-639. doi:10.1038/nature01335. PubMed: 12571597. 12571597

[76] Mohler PJ, Splawski I, Napolitano C, Bottelli G, Sharpe L et al. (2004) A cardiac arrhythmia syndrome caused by loss of ankyrin-B function. Proc Natl Acad Sci U S A 101: 9137-9142. doi:10.1073/pnas.0402546101. PubMed: 15178757. 15178757 PMC428486

[77] Adsit GS, Vaidyanathan R, Galler CM, Kyle JW, Makielski JC (2013) Channelopathies from mutations in the cardiac sodium channel protein complex. J Mol Cell Cardiol 61: 34-43. doi:10.1016/S0735-1097(13)60035-2. PubMed: 23557754. 23557754 PMC3720718

[78] Herren AW, Bers DM, Grandi E (2013) Post-translational modifications of the cardiac Na channel: contribution of CaMKII-dependent phosphorylation to acquired arrhythmias. Am J Physiol Heart Circ Physiol 305: H431-H445. doi:10.1152/ajpheart.00306.2013. PubMed: 23771687. 23771687 PMC3891248

[79] Aurlien D, Leren TP, Taubøll E, Gjerstad L (2009) New SCN5A mutation in a SUDEP victim with idiopathic epilepsy. Seizure 18: 158-160. doi:10.1016/j.seizure.2008.07.008. PubMed: 18752973. 18752973

